# The Use of Viatorr Stent at the Thoracic Outlet to Maintain Hemodialysis Access Function

**DOI:** 10.1177/15385744231183490

**Published:** 2023-06-13

**Authors:** Mohammad Ghasemi-Rad, Lauren Do, Michael Collard, Jai Cui, Zubin Irani

**Affiliations:** 1 Department of Radiology, Section of Vascular and Interventional Radiology, Baylor College of Medicine, Houston, TX, USA; 2 MD candidate, Baylor College of Medicine, Houston, TX, USA; 3 Department of Radiology, Section of Vascular and Interventional Radiology, Johns Hopkins All Childrens Hospital, St Petersburg, FL; 4 Department of Radiology, Section of Vascular and Interventional Radiology, Massachusetts General Hospital, 1811 Harvard Medical School, Boston, MA, USA; 5 Department of Radiology, Section of Vascular and Interventional Radiology, Massachusetts General Hospital, 1811 Harvard Medical School, Boston, MA, USA

**Keywords:** viatorr stent, thoracic outlet, hemodialysis access, central venous

## Abstract

**Purpose:**

Venous steno-occlusive disease at the thoracic outlet affects up to 30% of the hemodialysis population [1] causing arm swelling and hemodialysis access dysfunction. Balloon angioplasty in this region can be of limited utility given the rigid compressive effect of surrounding musculoskeletal (MSK) structures. Outcomes of using the Viatorr endoprosthesis (Gore Viatorr TIPS Endoprosthesis, Gore, Flagstaff AR, USA, Viatorr ®) within this region to salvage the HD access in patients who presented with dialysis access dysfunction is presented.

**Methods:**

A retrospective chart review was performed of our tertiary and quaternary care hospital system. Hemodialysis patients were included in the study if they were using an upper extremity arteriovenous fistula or graft for access, had a Viatorr stent placed in the central (subclavian and/or brachiocephalic) veins, and had follow up.

**Results:**

A total of nine patients were identified to meet the inclusion criteria. Four interventions were due to refractory lesions of the subclavian or brachiocephalic veins, and the other five interventions were for hemodynamically significant lesions refractory to angioplasty alone, all resulting in access dysfunction. Primary patency ranged from 36-442 days (geometric mean 156.6 days, range 19-442 days). No stent fracture was identified on imaging at any point during follow-up of these patients out to a maximum of 2912 days (Average 837 days).

**Conclusions:**

The Viatorr stent graft used in the HD population for clinically significant lesions at the thoracic outlet (TO) showed no structural failures (fractures) in this cohort.

## Background

Classic venous thoracic outlet syndrome (VTOS) is compression of the nerves, vein, or artery at the level of the thoracic outlet between the first rib, clavicle, and scalene muscles. The classic description is mostly in younger population and can present with venous thrombosis or intermittent symptomatic obstruction. Compression of neurologic structures is most common with a relative incidence of 80%, followed by venous (19%) and arterial (2%) [1]. Although the same anatomic predisposition can exist in dialysis patients, there is an added feature that is not present in the classic VTOS patients; namely the increased flow (venous return) from the upper extremity because of the HD acces which dilates the venous structures. This flow related venous expansion can then bring the venous wall into more exaggerated contact up against the surrounding structures and so replicate a VTOS-like picture affecting the subclavian vein. Increased flow also results in more shear stress on venous wall with scaring and stenosis of central and peripheral veins. This subclavian venous occlusive disease poses a continuing and significant burden to patients undergoing long-term hemodialysis, causing ipsilateral limb swelling, and potentially access dysfunction related to venous hypertension.^1^ The incidence of this VTOS like condition in hemodialysis patients has been increasing, so efficient management of this condition is essential [1]. Dialysis-related central stenosis can be due to multiple contributing factors, including catheter placement, venous flow, and compressive anatomy, leading to upper extremity HD access dysfunction.

Surgical decompression by first rib resection is the definitive treatment of classic VTOS, with adjunctive therapies to address the vein being compressed (e.g. venous angioplasty). However, most patients with dialysis have multiple underlying health conditions and are not candidates for decompressive surgery. Traditionally, non-covered stents have been used at this location. Stent placement is only considered if the angioplasty fails in a shorter time than expected (less than 3 months per KDOQI). Fractures of non-covered stents in this location can be due to continued compression force from the surrounding structures. The wire gauge of the Viatorr could offer better durability against the mechanical compression in the region of thoracic outlet, improving the efficacy of stenting at this site. In this study, we retrospectively reviewed the outcomes of hemodialysis patients who presented with clinically significant subclavian vein occlusion at the level of thoracic outlet that were treated with a Viatorr stent.

## Material and Methods

A retrospective chart review was performed of our tertiary and quaternary care hospital system. Hemodialysis patients were included if they were using an upper extremity arteriovenous fistula or graft for access and had a Viatorr stent (Gore, Flagstaff AR, USA) placed in the central (subclavian and/or brachiocephalic) veins and had follow-up post-placement including access usability and relief of symptoms such as arm tightness/swelling. Demographic, clinical, procedural, and imaging data were retrieved from the electronic medical record and picture archiving and communication system (PACS). Images were reviewed by both an interventional radiology fellow and an attending physician. Data was collected and analyzed using Microsoft Excel, and the geometric mean and range were reported. The institutional review board approved the study. Due retrospective nature of our study, informed consent was not obtained.

## Results

**Figure 1.**
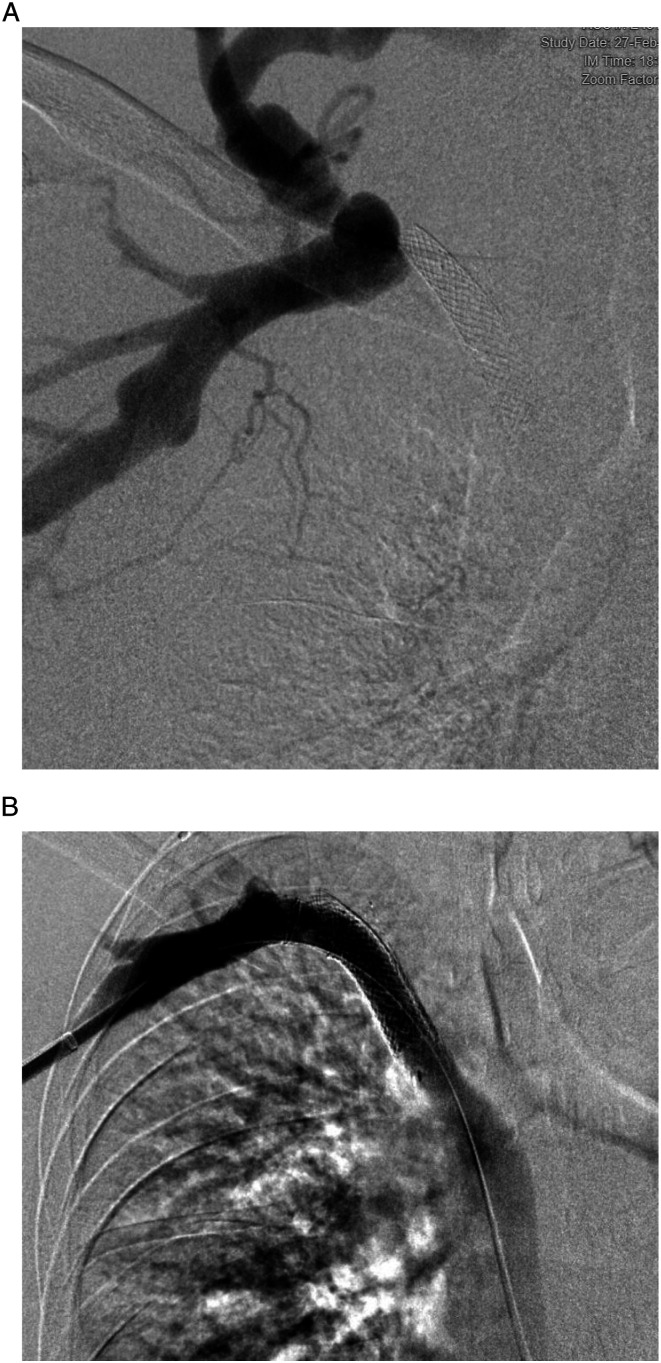
(A) pre-procedure digital subtraction venogram of right upper extremity demonstrated complete occlusion of right subclavian vein at the level of wall stent. (B) Digital subtraction venogram following venous recanalization and Viatorr stent placement demonstrated significant luminal gain with good forward flow.

A total of nine patients with malfunctioning dialysis fistula referred to our tertiary referral hospital were identified to meet the inclusion criteria. All patients had an AV fistula; no patients with an AV graft were identified for the study. There were 3 females and 6 males with an average patient age of 65.5 years at the time of stent placement. All patients presented with AVF malfunction and none with thrombosis. Per KDOQI guidelines, any patient presenting with recurrent central venous occlusion within 3 months is a candidate for stent placement. Four were placed due to recurrent occlusions of the subclavian or brachiocephalic veins, with the other five placed for hemodynamically significant stenosis, all resulting in access dysfunction refractory to angioplasty alone. Covered segment length ranged from 5-6 cm with the final diameter after stent angioplasty ranging from 8-10 mm. All stent placements were technically successful with brisk central flow at procedure completion, resulting in a 100% success rate with no immediate complications. Query revealed no immediate failures of Viatorr placement, and there were no cases in which Viatorr was pulled into the case and not used. All patients had a favorable radiological outcome, defined by less than 30% stenosis after treatment. All patients had symptomatic relief after stent placement with successful dialysis following the procedure. The primary patency, defined as the time between treatment and first intervention, ranged from 36-442 days in our patients (geometric mean 156.6 days, Range 19-442 days). One patient was lost on follow-up. Dialysis access was abandoned in one patient due to central occlusion, with two accesses later abandoned due to pseudoaneurysm 272 and 131 days after stent placement. Four patients died during follow up and two had a renal transplant. One had frequent thrombosis of access of unclear etiology (36,42, 121, and 130 days) and died from sepsis 4 months after the last intervention. One patient had prior stenosis that was treated with a wall stent (Figure 1). The geometric mean of duration of functional access between interventions was 194.2 days (Table 1). Any imaging that was available up to the time of review was scrutinized for stent location and integrity. No stent fracture or damage was identified at any point in imaging follow-up of these patients out to a maximum of 2912 days (Average 837 days).

**Table 1.** Demographic, Type of Fistula, Patient Characteristics.

Patient	Access Side	Access Age (Months)	Time to Primary patency (days)	Type of Fistula	Stent Location	Stent diameter (mm)
1	Right	52	19	Right brachiocephalic	Cephalic vein to SVC	10
2	Right	34	120	Right brachiocephalic	SCV	10
3	Right	52	124	Right brachiocephalic	BCV	10
4	Right	104	442	Right brachio-axillary	SCV, uncovered in BCV	10
5	Right	16	36	Right brachiocephalic	SCV, uncovered in BCV	10
6	Right	70	290	Right brachiocephalic	Uncovered in SCV, ending in proximal SVC	10
7	Right	96	150	Right brachiocephalic	BCV, uncovered in SCV	12
8	Left	3	335	Left brachiocephalic	BCV, uncovered at SCV/IJ confluence	12
9	Left	6	105	Left brachiocephalic	BCV, uncovered at BCV confluence	8

## Discussion

Stent placement within the subclavian arteries and veins has been shown to have poor durability, likely due to mechanical motion within this region causing repetitive stress and structural failure of the stent.^2^ Generally, stent placement in the subclavian vessels is not recommended.^3^ This is particularly problematic in dialysis patients who rely on patent outflow veins for their dialysis access and are often not good surgical candidates for definitive decompressive surgery.

### Stenosis Section

The incidence of central venous stenosis in hemodialysis access-related patients is between 20-40%. There is an especially high incidence of central venous stenosis in patients who have previously had subclavian catheters, potentially due to venous endothelial trauma and inflammation (3). Current KDOQI recommendations have PTA as first-line treatment for treating stenotic lesions, and if stenosis is persistent (>30% residual) or recurs within 3 months, then stent placement can be considered. If stenting is deferred, then frequent PTA or moving to another access site are other potential management strategies.

A study by Chen et al. used stenting for central venous stenosis in dialysis patients. Seventy-one patients were included in the study. 22 patients underwent stent placement with covered stent and 49 with bare stent.^1^ The use of a covered stent, a larger size vein (>12 mm), and the absence of vascular calcification were associated with a better patency rate.^1^ These results indicate that covered stents may improve patency rates for dialysis patients. In a study by Kundu et al, the patency of polytetrafluoroethylene (PTFE)-covered stent (encapsulated nitinol stent) was evaluated in the treatment of central venous stenosis in 14 patients, 3 of which were at the thoracic outlet (Bard Peripheral Vascular, Tempe, AZ).^4^ The study recorded primary patency rates at 3, 6, and 9 months of 100%, demonstrating the high efficacy of a PTFE-covered stent at the level of the thoracic outlet.^4^

Bare metal stents (BMS) are associated with a high degree of restenosis due to exposure to endothelium. They may limit future surgical revision and endovascular options.^1^ They also run the risk of fracture, migration, foreshortening, and most importantly, inciting intimal hyperplasia with recurrent stenosis.^4^ The covered stents are less susceptible to endothelial ingrowth due to PTFE covering, as previously evidenced. However, they can be associated with high rates of stenosis at the proximal and distal junctions with native veins, presenting a different problem.^5^

In comparing stenting to angioplasty, previous literature has shown little to no difference between bare metal stents and angioplasty in the treatment of venous stenosis. A study by Bakken et al. compared primary balloon angioplasty with primary bare metal stenting (BMS) in patients with dialysis AVF-related central stenosis There was no significant difference in overall patency results between the PTA and the BMS group.^6^

The Viatorr Endoprosthesis is a stent made of outer self-expanding nitinol with .2 mm thickness having a zig-zag spiral configuration. The inner side is covered with three layers of ePTFE with different porosities designed to resist bile permeation. The stent was originally made for a trans-jugular intrahepatic portosystemic shunt (TIPS) for this purpose. It has a two-cm uncovered section and varied lengths of the covered portion.^7,8^ The choice of Viatorr stent use at the level of the thoracic outlet was based primarily on the thicker wire gauge used in the scaffold. The covered component was also felt to offer a potential benefit given the BMS outcomes in this HD circuit. The ipsilateral internal jugular vein was occluded in these patients so ‘jailing’ off the jugular vein was not a concern, and patients in this study were previously deemed too sick to undergo first rib resection, leaving stenting as their most viable (and potentially durable) treatment option.

The outcomes of this cohort was that all patients had angiographic and anatomically successful stent placement (less than 30% stenosis after treatment). These results are consistent with previous findings that covered stent placement can lead to better long-term patency rates for dialysis patients. Covered stent placement is a viable treatment option, especially for those experiencing venous stenosis due to hemodialysis treatment or who are not candidates for surgical intervention. Major disadvantages of covered stents include high cost and covering collateral or major veins during deployment of the stent. However, in the case of Viatorr stent, which has a 2cm uncovered segment, can avoid jailing any important branches and preserve some collateral veins.

Antiplatelet therapy after stent graft placement may be of concern. Kundu et al. did not prescribe patients any anti platelet therapy after placement of a covered stent with a result of 100% patency at 9 months [3]. Rajendran et al. only prescribed long-term oral anticoagulation when patients presented with signs of venous thrombosis [6]. In this study, we did not any added anticoagulation.

Disadvantages of this study include a small sample size with one patient lost to follow-up. Follow-up was also conducted retrospectively and was therefore not as comprehensive as would be a prospective study.

## References

[1] Chen B Lin R Dai H , et al. One-year outcomes and predictive factors for primary patency after stent placement for treatment of central venous occlusive disease in hemodialysis patients. Ther Adv Chronic Dis. 2022;13:20406223211063039. doi:10.1177/20406223211063039 35198135 PMC8859657

[2] Schropp L de Kleijn R Vonken EJ , et al. Multicenter case series and literature review on durability of stents in the thoracic outlet. J Endovasc Ther. 2023;30:355-363. doi:10.1177/15266028221081078 35255758

[3] Illig KA Rodriguez-Zoppi E Bland T Muftah M Jospitre E . The incidence of thoracic outlet syndrome. Ann Vasc Surg. 2021;70:263-272. doi:10.1016/j.avsg.2020.07.029 32771464

[4] Kundu S Modabber M You JM Tam P Nagai G Ting R . Use of PTFE stent grafts for hemodialysis-related central venous occlusions: Intermediate-term results. Cardiovasc Intervent Radiol. Oct 2011;34(5):949-957. doi:10.1007/s00270-010-0019-4 21069331

[5] Lok CE Huber TS Lee T , et al. KDOQI clinical practice guideline for vascular access: 2019 update. Am J Kidney Dis. 2020;75(4 Suppl 2):S1-S164. doi:10.1053/j.ajkd.2019.12.001 32778223

[6] Bakken AM Protack CD Saad WE Lee DE Waldman DL Davies MG . Long-term outcomes of primary angioplasty and primary stenting of central venous stenosis in hemodialysis patients. J Vasc Surg. 2007;45(4):776-783. doi:10.1016/j.jvs.2006.12.046 17398386

[7] Cejna M Peck-Radosavljevic M Thurnher S , et al. ePTFE-covered stent-grafts for revision of obstructed transjugular intrahepatic portosystemic shunt. Cardiovasc Intervent Radiol. Sep-Oct 2002;25(5):365-372. doi:10.1007/s00270-001-0121-8 11981612

[8] Hausegger KA Karnel F Georgieva B , et al. Transjugular intrahepatic portosystemic shunt creation with the Viatorr expanded polytetrafluoroethylene-covered stent-graft. J Vasc Intervent Radiol. 2004;15(3):239-248. doi:10.1097/01.rvi.0000116194.44877.c1 15028808

